# Effect of Bone Cement Thickness on the Risk of Scalded Skin in Joint Surgery

**DOI:** 10.1111/os.13700

**Published:** 2023-04-24

**Authors:** Binglong Li, Kaifei Han, Yang Yu, Junyi He, Houyi Sun, Qunshan Lu, Lei Li, Tong Zheng, Baoqing Zhang, Peilai Liu

**Affiliations:** ^1^ Department of Orthopedics Qilu Hospital of Shandong University Jinan China; ^2^ Cheeloo College of Medicine Shandong University Jinan China; ^3^ Department of Pathology Qilu Hospital of Shandong University Jinan China

**Keywords:** Arthroplasty, Bone Cement, Orthopaedic, Scald, Skin

## Abstract

**Objective:**

Bone cement releases a large amount of heat as it polymerizes. Skin burns caused by discarded bone cement are not well understood during arthroplasty. It is important to study the correlates and mechanisms of scalding and to accurately evaluate the severity of burns to guide treatment decisions.

**Methods:**

Standardized burns were created in eight anesthetized rabbits using different thicknesses of bone cement. Bone cement was uniformly stirred to make thicknesses of 1 mm, 4 mm, 8 mm, 12 mm, 16 mm, and 20 mm and a 20 × 40 mm cuboid. Bone cement samples were then placed on the back of a rabbit, and the temperature changes were recorded with an industrial digital thermometer. One hour later, the appearance of scalded skin was observed, and the rabbits were euthanized. The scalded parts were cut to make pathological sections and stained with HE, and the differences in the depth of the scalded skin caused by different thicknesses of bone cement were observed under a light microscope.

**Results:**

Damage caused by 1 mm‐, 4 mm‐, 8 mm‐, 12 mm‐, 16 mm‐, and 20 mm‐thick bone cement samples mainly involved the epidermis, the papillary dermis, the reticular dermis layer, and the full thickness of the skin and the subcutaneous tissue. The maximum temperature of 1 mm, 4 mm, 8 mm, and 12 mm bone cementation had a statistically significant difference (*p* < 0.001), while there was no significant difference between 12 mm, 16 mm, and 20 mm samples (*p* = 0.856). The time to severe scalding with bone cement at temperatures above 70°C was significantly different between different thicknesses (*p* < 0.001).

**Conclusion:**

The heat released by different thicknesses of bone cement leads to different maximum temperatures and the duration of severe burns, resulting in different degrees of skin burns. Attention should be paid to discarded bone cement to prevent this potential complication in knee arthroplasty.

## Graphical abstract

The maximum temperature and the duration of severe burns caused by different thicknesses of bone cement are different, resulting in different degrees of skin burns. For better diagnosis and treatment, surgeons should combine the clinical situation with the pathological observation of scald. Care should be taken to discard excess bone cement promptly to prevent this potential complication. (A) The measured method and instruments; (B) Appearance of skin scalded by different thicknesses of bone cement (12 mm and 16 mm); (C) Pathological features of scalded skin with bone cement, the arrow represents vacuolar degeneration of nerve fibers and hyperemia of blood vessels in the reticular layer, and collagen fiber aggregation, degeneration; (D) The change in temperature with time in different thickness of bone cement

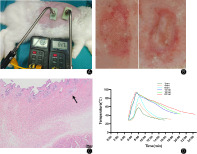

## Introduction

Knee osteoarthritis is the most common chronic degenerative joint disease in the elderly.^1^ Currently, knee arthroplasty is an effective treatment for improving the quality of life of patients with end‐stage osteoarthritis. Polymethyl methacrylate (PMMA), commonly known as bone cement, is widely used in various subspecialties of orthopaedics, including fixation of implants in total knee arthroplasty and unicompartment knee arthroplasty. After mixing, the liquid monomer polymerizes around the prepolymerized powder to form a dough‐like plastic mass, which is placed as an anchoring agent between the bone and the implant. Bone cement is self‐setting but has no adhesive properties. There is no chemical connection between the bone and the prosthesis, but good fixation of the bone and prosthesis is achieved through microinterlocking and bulk‐filling mechanisms.^2^, ^3^, ^4^ The solidification process of bone cement goes through four stages—mixing, waiting, application, and setting—to form hardened bone cement so that the prosthesis can be stably fixed.^5^

The bone cement polymerization process is an exothermic reaction that releases a large amount of heat. Bone cement can have a damaging thermal effect on bone, bladder, blood vessels, and nerves.^6^, ^7^, ^8^ Murayama et al.^9^ showed that excess discarded bone cement that stuck to the bandage resulted in scalding of the calf, lateral side of the heel, and lateral and medial side of the femur during total hip arthroplasty. Skin burns caused by the thermal effect of discarded bone cement are a rare complication in orthopaedics. In the knee arthroplasty, bone cement placed between the osteotomy surface and the prosthesis was squeezed, scraped, and adhered to the calf bandage, which resulted in skin burns at heel or calf in the knee arthroplasty.

Bone cement burns are characterized by protein coagulation and tissue necrosis. Thermal injury can alter endothelial cell actin reorganization and contraction, causing mast cells to release bradykinin, which leads to vasodilation and increased permeability.^10^ The thermal effect of bone cement induces pro‐inflammatory mediators such as IL‐1, IL‐6, and TNF‐α activates neutrophils and monocytes, and induces apoptosis in the scalded area.^11^ Therefore, bone cement burns destroy the natural skin barrier, leading to local blood circulation disorders and providing good conditions for bacterial invasion.^12^ Bone cement‐related skin burns have not received enough attention in clinical practice, resulting in patients not receiving timely and effective treatment after intraoperative burns. There have been reported cases of skin scalded by bone cement,^2^, ^9^, ^13^ but there is a lack of research on the correlates and mechanisms of skin scalded by bone cement.

The purpose of this study was (i) to investigate the effect on the severity of scalded skin with different thicknesses of discarded bone cement, (ii) to remind orthopaedic surgeons to prevent the occurrence of bone cement‐induced scalding, and (iii) to guide surgeons to choose appropriate treatments.

## Methods

### Ethics Statement

This experiment was ethically approved by the Animal Ethics Committee of Qilu Hospital of Shandong University (IACUC Issue NO. DWLL‐2022‐014). All operations and animal care in this experiment complied with the national standards and guidelines for the use and management of laboratory animals formulated by the National Standardization Management Committee.

### Animal Handling, Sedation, Analgesia, and Anesthesia

Eight conventional (CV) rabbits aged 8 weeks and weighing 2.5 to 3 kg from Jinan Jinfeng Experimental Animal Co. Ltd. were used in this experiment. To adapt to the environment, these experimental rabbits were fed individually in the animal laboratory for 1 week before the start of the experiment. Standard food was provided regularly, and water was freely available. The animals lived under a light–dark cycle of 12 hours of light followed by 12 hours of darkness. Rabbits fasted overnight before general anesthesia. Rabbits were anesthetized by intramuscular injection of atropine 0.05 mg/kg, diazepam 1 mg/kg, and ketamine 0.5 ml/kg. Sustained general anesthesia was achieved by mask inhalation of 2% isoflurane at a rate of 0.4 mL/min through Ayre's tee at 4 L/min fresh gas flow. An intraperitoneal injection of buprenorphine 0.1 mg/kg was used for analgesia. The flanks and backs of the rabbits were removed with scissors, the skin was scrubbed with soapy water, and short hairs were removed with a depilatory cream.

### Study Design and Burn Creation

**Fig. 1:**
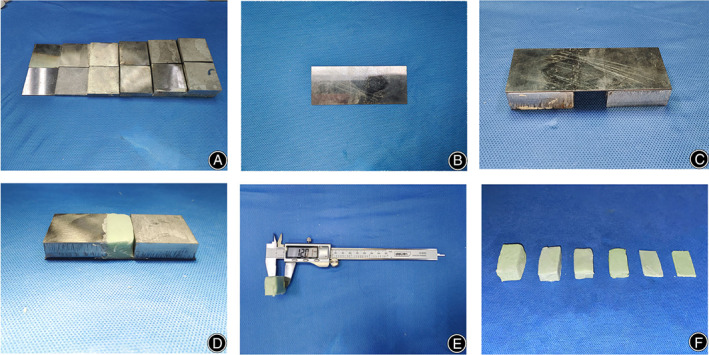
Bone cement samples and research materials. (A) 40 × 40 mm iron blocks with heights of 1 mm, 4 mm, 8 mm, 12 mm, 16 mm, and 20 mm. (B) An iron plate of 100 × 40 × 1 mm. (C) Research materials of 40 × 40 × 12 mm bone cement cube. (D) The production process of 40 × 40 × 12 mm bone cement sample. (E) Bone cement sample of 40 × 40 × 12 mm. (F) Different thickness of bone cement

The severity of tissue damage after bone cement scalding was investigated in eight rabbits. Using PALACOS R + G bone cement (Heraeus Medical GmbH, Wehrheim, Germany), the liquid monomer was poured into a bowl, the polymer powder was added, and the mixture was stirred uniformly for 1 minute. When the dough‐like bone cement did not stick to the rubber gloves, it was measured with a digital Vernier caliper (DL91150, Deli Group Co. Ltd., Zhenjiang, China). We customized two for each 40 × 40 mm iron block with heights of 1 mm, 4 mm, 8 mm, 12 mm, 16 mm, and 20 mm (Figures 1A and S1), and a 100 × 40 × 1 mm iron plate (Figure 1B). Two 40 × 40 × 12 mm iron blocks and a 100 × 40 × 1 mm iron plate were placed on it, with the edges aligned, so that the gap was 40 × 20 mm and the thickness was 12 mm (Figure 1C). Place the bone cement between the two iron blocks, place the iron plate on the iron blocks, align the two ends, squeeze, and use a scalpel to cut off the excess bone cement along the edge to make a 40 × 40 × 12 mm bone cement (Figure 1D,E). Cubes of 20 × 40 mm with heights of 1 mm, 4 mm, 8 mm, 16 mm, and 20 mm were made by the same method (Figure 1F). The control group was normal skin without scald. Bone cement samples of different thicknesses were placed on the back skin of rabbits. An industrial digital thermometer (DM6802A dual channel thermometer, Lihua Instruments Company, Shenzhen, China) was placed in the central part of the bone cement (Figure S2A,B), and the temperature was recorded every 20 s for 5 min until the temperature fell steadily below 44°C. Next, the bone cement was placed on the skin for 20 min and removed, and the appearance of the skin was observed 1 h later. On each of the eight rabbits, we created four burns. Two bone cement burns were created on each side of the back of each rabbit (Figure S2C). We created five replicates of each thickness of bone cement. In this experiment, rectangular bone cement scalding perpendicular to the axis of the spine was evaluated.^14^, ^15^ The skin under the center of the bone cement was used for pathological analysis. When assessing the severity of tissue damage, the pathologist was blinded to the grouping.

The experimental rabbits were euthanized with 200 mg/kg sodium pentobarbital. A 20 × 40 mm patch of the scalded part skin of the rabbit was excised, and a small piece was cut from the middle part of the excised skin. The scalded skin was fixed with 10% formalin, and then pathological sections of the scalded skin were made. HE staining and light microscopy was used to observe the pathological changes in the scalded skin in each group.

### Outcomes

The primary outcome of the study was the depth of the burn as determined by histopathological assessment. The depth of burn was mainly determined by the depth of collagen fiber agglutination and degeneration combined with the maximum depth of damaged vascular endothelial cells or erythrocyte disintegration. Secondary outcomes were skin appearance, the average maximum temperature of bone cement, and duration of exposure to temperatures above 70°C.^16^, ^17^

### Data Analysis

The data were analyzed by SPSS V26.0 (International Business Machines Corporation, Arizona, USA). Data that conformed to a normal distribution are expressed as the mean ± standard deviation, and those that did not conform to a normal distribution are expressed as the median and interquartile range. Analysis of variance was used to compare the differences between groups. If it found a difference, the Student–Newman–Keuls (SNK) test was used for pairwise comparison, and the Kruskal–Wallis rank sum test was used to compare nonparametric data. *p* < 0.05 was considered statistically significant.

## Results

### Histopathology

**Fig. 2:**
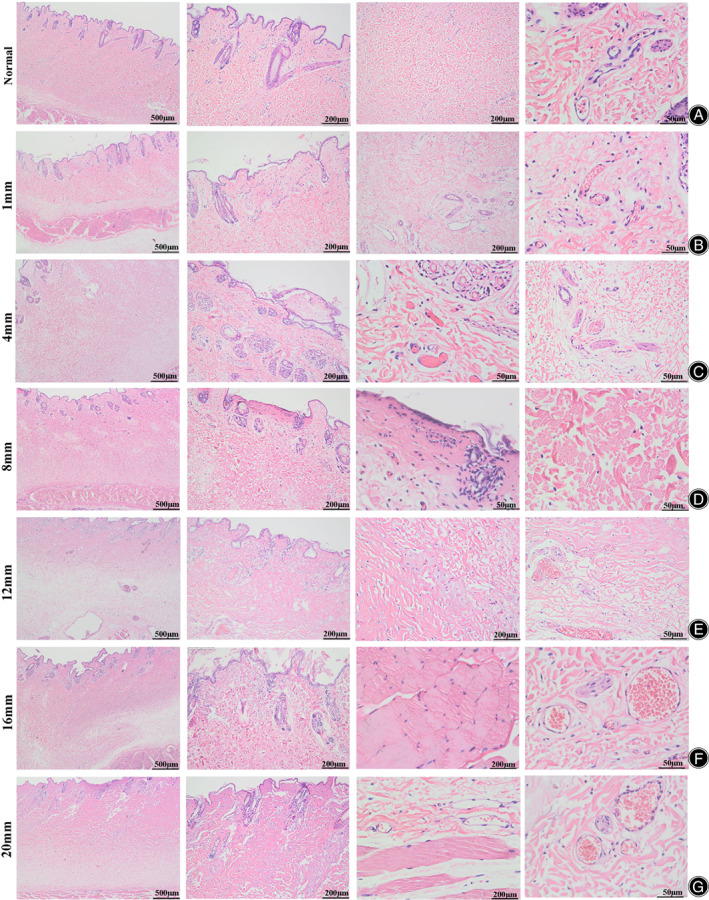
Pathological features of scalded skin with different thicknesses of bone cement. (A) Normal skin. (B) Subepidermal fissures, and vascular congestion in the dermal papilla layer. (C) Subepidermal fissures and hair follicle vacuolar degeneration, intravascular erythrocyte disintegration, and degeneration in the dermal papilla layer. (D) Basal layer vacuolar degeneration, subepidermal fissure, papillary layer collagen fiber aggregation, degeneration, focal nucleus debris around necrosis, and disordered reticular layer collagen fibers. (E) Epidermis basal layer vacuolar degeneration, subepidermal fissure; collagen fiber aggregation and degeneration, vascular congestion, and nerve fiber vacuolar degeneration in the papillary layer and reticular layer. (F) Epidermal subepidermal fissure, partial epidermal necrosis, and exfoliation; vacuolar degeneration of nerve fibers and hyperemia of blood vessels in the reticular layer. (G) Diffuse subepidermal fissure, partial epidermal necrosis and exfoliation; vascular endothelial swelling, neurofibrillary vacuolar degeneration; skeletal muscle interstitial edema, and increased gaps

As shown in Figure 2A, in normal skin, the collagen fibers in the epidermis and dermis were arranged in an orderly manner; skin appendages, such as hair follicles and sweat glands, could be seen in the dermis; and normal blood vessels and nerve fibers could be seen. As shown in Figure 2B, some epidermal basal layer cells were vacuolated, and cracks were observed under the epidermis in 1‐mm‐thick bone cement. As shown in Figure 2C, compared with 1‐mm‐thick samples, collagen fibers with 4‐mm‐thick bone cement‐scalded skin were more disordered and agglutinated, more hair follicles were vacuolated, and red blood cells were disintegrated in the dermal papilla layer. As shown in Figure 2D, 8‐mm‐thick bone cement scalded the skin involving the reticular dermis. Focal necrosis was seen in the papillary layer, surrounded by infiltrating inflammatory cells, nuclear disintegration, and nuclear debris. Collagen fibers in the reticular layer were disordered, degenerated, and broken up focally. As shown in Figure 2E, dermal papillary and reticular scalding was more severe with 12 mm of bone cement. Capillary occlusion was seen in the papillary layer. Reticular edema was evident, with vacuolar degeneration of nerve fibers and vascular congestion. As shown in Figure 2F, fibrotic aggregation and degeneration caused by 16‐mm bone cement involve subcutaneous loose connective tissue. Part of the epidermis was necrotic and exfoliated. We saw arrectores pilorum degeneration in the papillary layer, vacuolar degeneration of nerve fibers, and swelling of vascular endothelial cells in the reticular layer. As shown in Figure 2G, skin scalded by 20 mm of bone cement involved the whole layer. The subcutaneous loose connective tissue was oedematous and was surrounded by inflammatory cells. Most of the blood vessels in the whole layer were occluded, the vascular endothelial cells of the remaining vessels were swollen, and the nerve fibers showed vacuolar degeneration. Skeletal muscle interstitial edema increased with the gap.

The depth of skin burns caused by 1 mm, 4 mm, 8 mm, 12 mm, 16 mm, and 20 mm thickness of bone cement was 24.5 ± 3.7 μm, 380.0 ± 90.8 μm, 1200.0 ± 209.2 μm, 1400.0 ± 271.0 μm, 3300.0 ± 326.0 μm, and 3350.0 ± 271.0 μm, respectively. Bone cement that was 16 mm and 20 mm thick involved the full thickness of the skin and subcutaneous tissue, thus the depth of skin burns was similar due to the limited thickness of rabbit skin.

### Skin Appearance

**Fig. 3:**
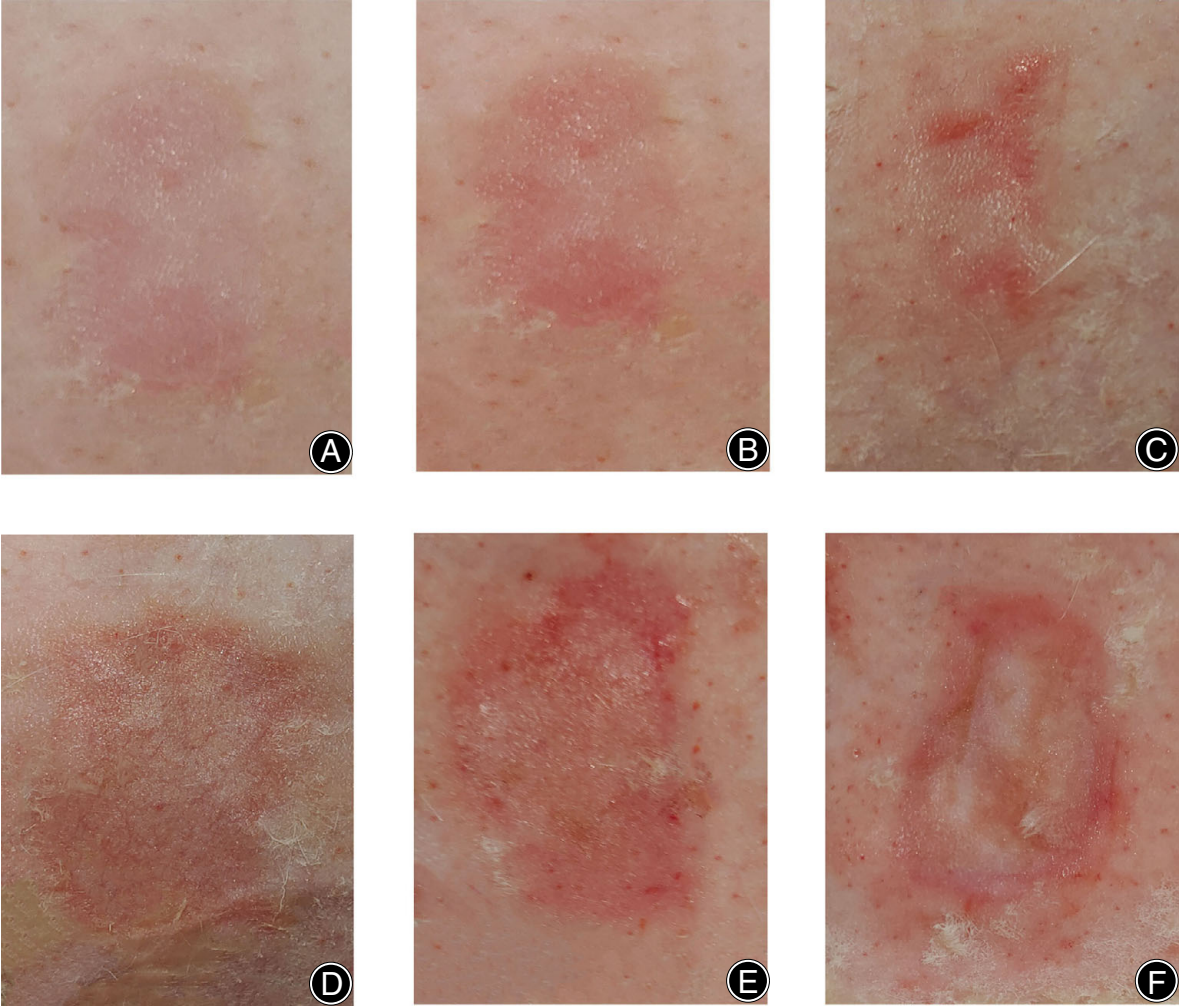
Appearance of rabbit skin 1 hour after bone cement burn. (A) Appearance of skin scalded skin by 1‐mm‐thick bone cement. (B) Appearance of skin scalded skin by 4‐mm‐thick bone cement. (C) Appearance of skin scalded by 8‐mm‐thick bone cement. (D) Appearance of skin scalded by 12‐mm‐thick bone cement. (E) Appearance of skin scalded by 16‐mm‐thick bone cement. (F) Appearance of skin scalded by 20‐mm‐thick bone cement

As shown in Figure 3A, skin burns caused by 1‐mm‐thick bone cement showed mild redness, swelling, and dryness of the skin. As shown in Figure 3B, skin scalded by 4‐mm‐thick bone cement was erythematous, dry, and slightly swollen. As shown in Figure 3C, the skin with 8‐mm‐thick bone cement had more redness and swelling than 4‐mm‐thick bone cement, and blisters of different sizes were visible. After removing the blisters, the wound surface was rosy, moist, and soft. As shown in Figure 3D, the skin at the site of 12‐mm‐thick bone cement showed obvious wound redness and swelling, the local skin was whitish, small blisters were formed that contained light‐yellow clear liquid, and the wound surface was slightly wet. As shown in Figure 3E, the skin with 16‐mm‐thick bone cement was red and white, with obvious edema, dry skin, and no exudate. As shown in Figure 3F, 20‐mm‐thick bone cement resulted in skin lesions with waxy white skin, focal burnt yellow or yellowish‐brown skin in the central area.

### Temperature

**Fig. 4:**
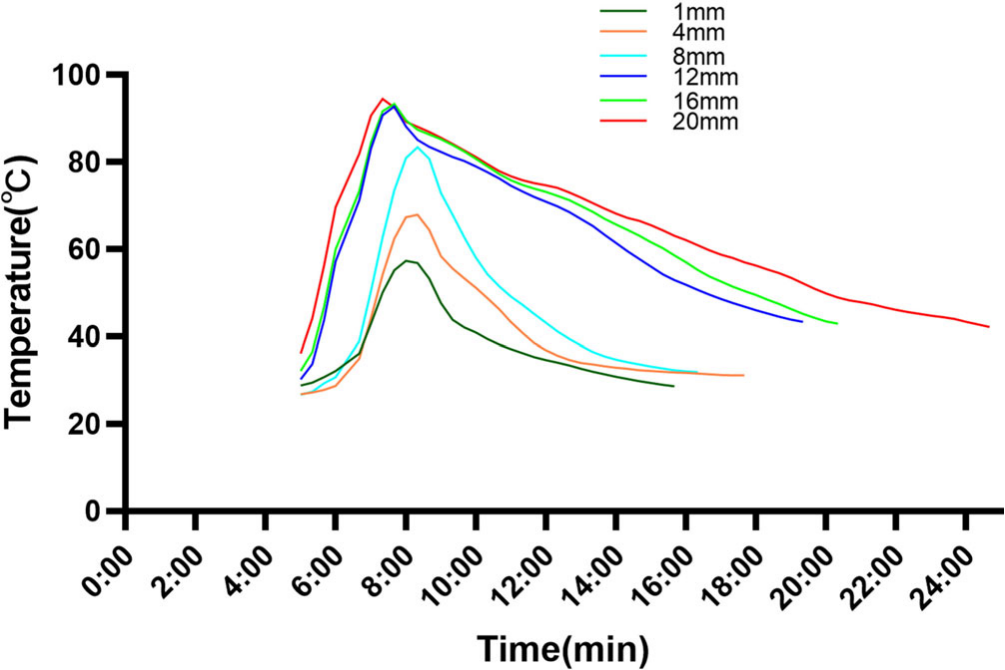
The change in temperature with time in different thickness groups

During the solidification process of bone cement, the average maximum temperature of bone cement with different thicknesses is shown in Table 1. Table 2 shows that in the process of PMMA polymerization, the maximum temperature of 1 mm, 4 mm, and 8 mm bone cement was significantly different from that of 12 mm, 16 mm, and 20 mm bone cement (*p* < 0.001), but the maximum temperature of bone cement with thicknesses of 12 mm, 16 mm, and 20 mm had no significant difference (*p* = 0.856). Figure 4 shows that the temperature changes over time from 5 minutes on.

**TABLE 1:** Comparison of the maximum temperature of bone cement with different thicknesses (°C)

			95% CI				*p* value
	Mean	SD	Upper limit	Lower limit	Maximum	Minimum	
1 mm	63.3	1.13	61.85	64.67	62.0	64.5	0.000
4 mm	73.5	1.94	71.07	75.89	70.4	75.6	
8 mm	87.9	1.89	85.51	90.21	85.7	90.7	
12 mm	93.0	1.38	91.30	94.74	91.0	94.8	
16 mm	93.0	1.50	91.16	94.88	91.9	95.4	
20 mm	93.2	1.29	91.60	94.80	91.8	95.3	

Abbreviations: CI, confidence interval; SD, standard deviation.

**TABLE 2:** SNK postmortem comparison of the maximum temperature of bone cement with different thicknesses

Thickness (mm)	Subaggregate of alpha = 0.05			
	1	2	3	4
1	63.3 (1.13)			
4		73.5 (1.94)		
8			87.9 (1.89)	
16				93.0 (1.38)
12				93.0 (1.50)
20				93.2 (1.29)
Sig.	1.000	1.000	1.000	0.982

*Note*: Values are presented as mean (SD); SNK, Student–Newman–Keuls test.

### Duration of Exposure to Temperatures above 70°C

Table 3 shows the duration of skin which was exposed to bone cement of different thicknesses at temperatures above 70°C. Table 4 shows that the thicknesses of bone cement were significantly correlated with the duration of exposure to temperatures 70°C and above during the polymerization process.

**TABLE 3:** Comparison of the duration for which different thicknesses of bone cement above 70°C (min)

			95% CI				*p* value
	Mean	SD	Upper limit	Lower limit	Maximum	Minimum	
1 mm	0.00	‐	‐	‐	‐	‐	0.000
4 mm	0.68	0.15	0.50	0.87	0.50	0.83	
8 mm	2.08	0.50	1.46	2.70	1.50	2.83	
12 mm	5.37	0.53	4.71	6.03	4.67	6.00	
16 mm	6.20	0.32	5.80	6.60	5.83	6.67	
20 mm	7.43	0.48	6.83	8.03	6.83	8.17	

Abbreviations: CI, confidence interval; SD, standard deviation.

**TABLE 4:** Postmortem comparison of the duration exposure to different thicknesses of bone cement above 70°C (S‐N‐K test)

Thickness (mm)	Subaggregate of alpha = 0.05					
	1	2	3	4	5	6
1	0.00					
4		0.68 (0.15)				
8			2.08 (0.50)			
16				5.36 (0.53)		
12					6.20 (0.32)	
20						7.43 (0.48)
Sig.	1.000	1.000	1.000	1.000	1.000	1.000

*Note*: Values are presented as mean (SD); SNK, Student–Newman–Keuls test.

## Discussion

### Analysis of Research Results

Our study demonstrates that with the increase of bone cement thickness, the burn depths increase with increasing the maximum temperature of bone cement and duration of severe burns. This is consistent with the study by Adam et al.^18^ Bone cement from 1 mm to 12 mm thick caused an increase in the maximum temperature and duration of exposure to scalding temperatures, with significant differences in scald severity. Although the maximum temperatures of 12 mm, 16 mm, and 20 mm thick bone cement were similar, the duration of exposure resulting in severe burns increased. Thus, the severity of skin burns was different.

In the field of arthroplasty, intraoperative bone cement‐related skin burns generally occur in the posterior ankle region and the middle and lower third of the posterior calf region. The reason may be that the discarded bone cement adhered to the calf bandage was still in the exothermic stage. After bone cement solidifies, bone cement contacts the patient's skin for a long time, resulting in skin burns. Due to the use of femoral nerve block in the operation and the use of postoperative analgesics, the patient's ankle pain was not obvious, which results in delayed discovery of the burn.^19^, ^20^ During hospitalization, the doctor was not fully aware of the scald, and the scalded site was not effectively treated, resulting in worsening after discharge.

### Relationship between Burn Degree and Thickness of Bone Cement

During the polymerization of 1 mm thick bone cement, skin scalding involves the skin epidermis, and it is a first‐degree scald. A sample of bone cement 4 mm thick causes exothermic damage to the skin epidermis and dermis papillary layer, which results in a partial thickness. Bone cement with thicknesses of 8 mm and 12 mm causes the skin below the papillary dermis layer, but remains part of the reticular layer, which was a full thickness. Bone cement that was 16 mm and 20 mm thick involved the full thickness of the skin and subcutaneous tissue, resulting in a third‐degree burn.

### Management of Different Degree of Burns

For better diagnosis and treatment, surgeons should combine the clinical situation with the pathological observation of scald. Since the skin barrier is intact in a first‐degree scald, there is generally no need for treatment. If the blisters are intact in a partial thickness, the blisters are not removed, and the blister fluid is extracted with an empty needle. If the blistered skin is avulsed, a sterile oil‐based dressing is used. Notably, frequent dressing changes should be avoided in the absence of wetting or other signs of infection to avoid damaging the regenerated epithelium.^21^, ^22^, ^23^ In a full thickness, wounds should be washed with soapy water to remove loose skin and blisters and treated with topical ointments to maintain a moist environment.^24^ Numerous studies have shown^25^ that silver sulfadiazine can affect skin re‐epithelialization, so it is not recommended for superficial wounds. Although escharotomy or scabbing and skin grafting are recommended for third‐degree burns,^22^, ^26^ due to the small scope of bone cement‐scalded skin, epithelial regeneration and repair of healthy skin at the edge of the wound can result in scar healing.

### Clinical Guiding Significance for Studying Thicknesses of Bone Cement and the Trend of Burn Duration

**Fig. 5:**
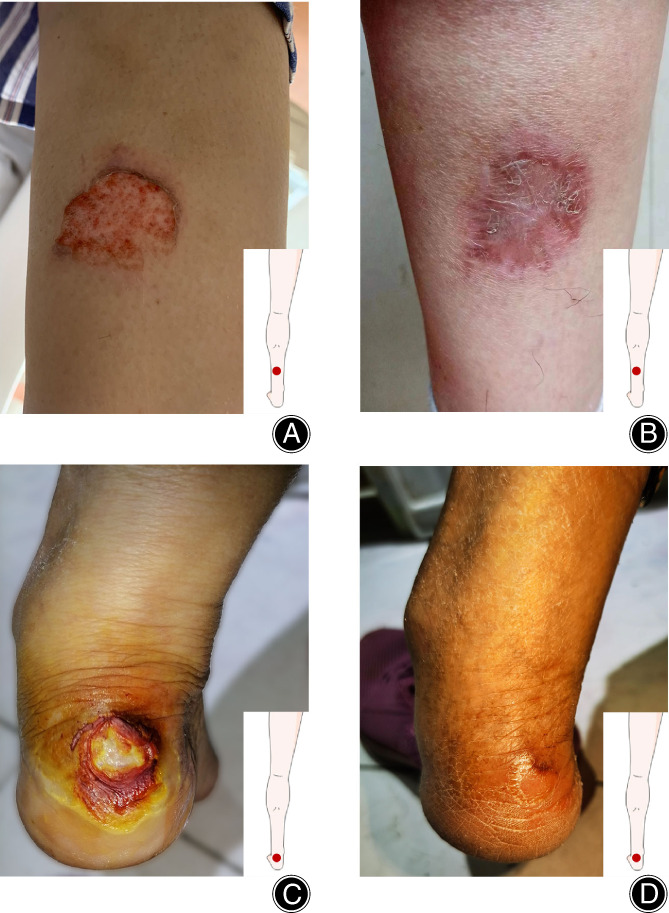
Appearance of cement‐scalded skin during knee arthroplasty. (A) The appearance of scalded skin in the middle and lower 1/3 of the posterior region of the calf on the first day after UKA in patient 1. (B) The recovery of the patient 1 at 1 month after surgery. (C) The skin damage experienced by patient 2 in the heel 3 days after surgery. (D) Patient 2 recovered 3 months after the operation, and the skin lesions were healed

Timely and accurate assessment of the severity of burns according to the characteristics of the burn is essential to guide the next treatment decisions. There were two cases of bone cement skin burns of different degrees in our hospital last year (Figure 5). Most discarded bone cement during the operation is between 3 to 10 mm thick, and a few can reach more than 12 mm. Therefore, once the prosthesis is placed, check whether the discarded bone cement adheres to the calf bandage to avoid continuous burns. Reduce the amount of bone cement during the operation, and pre‐cool the bone cement before the operation to slow down the polymerization reaction rate of the bone cement, which is beneficial to reduce the risk of bone cement burns. Orthopaedic surgeons should make clinical evaluations and empirical management of scalds based on the approximate range of thickness of discarded cement and skin appearance during the operation. During the operation, the surgeon and assistant should pay close attention to the whereabouts of the bone cement to proactively prevent the occurrence of bone cement burns. Training is regularly carried out to enhance the medical staff's awareness of scalding. Once scalding occurs, doctors should increase the awareness and education of patients when they are discharged from the hospital and follow them up regularly to prevent wound infection.

### Strengths and Limitations

This study innovatively evaluated the extent of skin scald caused by discarded bone cement and quantitatively assessed the degree of skin damage by measuring the temperature of bone cement and the thickness of skin scald. In addition, it could guide surgeons to choose appropriate treatments. However, our study has the following limitations. The preferred time point for assessing the depth of histological damage caused by scalding can be controversial.^27^, ^28^ In this study, we chose 1 hour after burn as the observation time point for assessing burn depth.^15^, ^29^ Another limitation of this study is that we did not consider the situation where the discarded bone cement first contacted deeper layers of skin, such as muscles and dermis. In the future, the effects of different treatments such as cold compress and scald ointment should be included. Further studies are needed to assess histopathological changes over time.

### Conclusion

With the increase of bone cement thickness, the maximum burn temperature and duration of severe burns increased, and thus the severity of skin scald increased. Attention should be paid to discarded bone cement to prevent this potential complication.

## Author Contribution

Binglong Li mainly completed the study design, data collection and analysis, and a draft of the manuscript. Kaifei Han completed the study design and a draft of the manuscript. Yang Yu completed the execution of the experiment. Junyi He completed pathological analysis. Houyi Sun and Qunshan Lu completed the draft of the manuscript. Tong Zheng and Lei Li completed data collection and analysis. Baoqing Zhang and Peilai Liu completed the study concept and study design. All authors read and approved the final manuscript.

## Conflicts of Interest

The authors have no relevant financial or non‐financial interests to disclose.

## Ethics Approval

This study was performed in line with the principles of the Declaration of Helsinki. Approval was granted by the Ethics Committee of Qilu Hospital of Shandong University (IACUC Issue NO. DWLL‐2022‐014).

## Supporting information


**Figure S1.** Iron blocks with heights of 1 mm, 4 mm, 8 mm, 12 mm, 16 mm, and 20 mm
**Figure S2.** The location of the cement scald. (A) The measured area in skin. (B) The measured area in bone cement. (C) The measured method and instruments
