# Supplementary material for: Effect of Bone Cement Thickness on the Risk of Scalded Skin in Joint Surgery
Source: Orthop Surg. 2023 Apr 24;15(6):1645–53. doi: 10.1111/os.13700 (PMC10235157; doi:10.1111/os.13700)
Supplement: Supplementary file 1 — Figure S1. Iron blocks with heights of 1 mm, 4 mm, 8 mm, 12 mm, 16 mm, and 20 mm Figure S2. The location of the cement scald. (A) The measured area in skin. (B) The measured area in bone cement. (C) The measured method and instruments [file OS-15-1645-s001.docx]

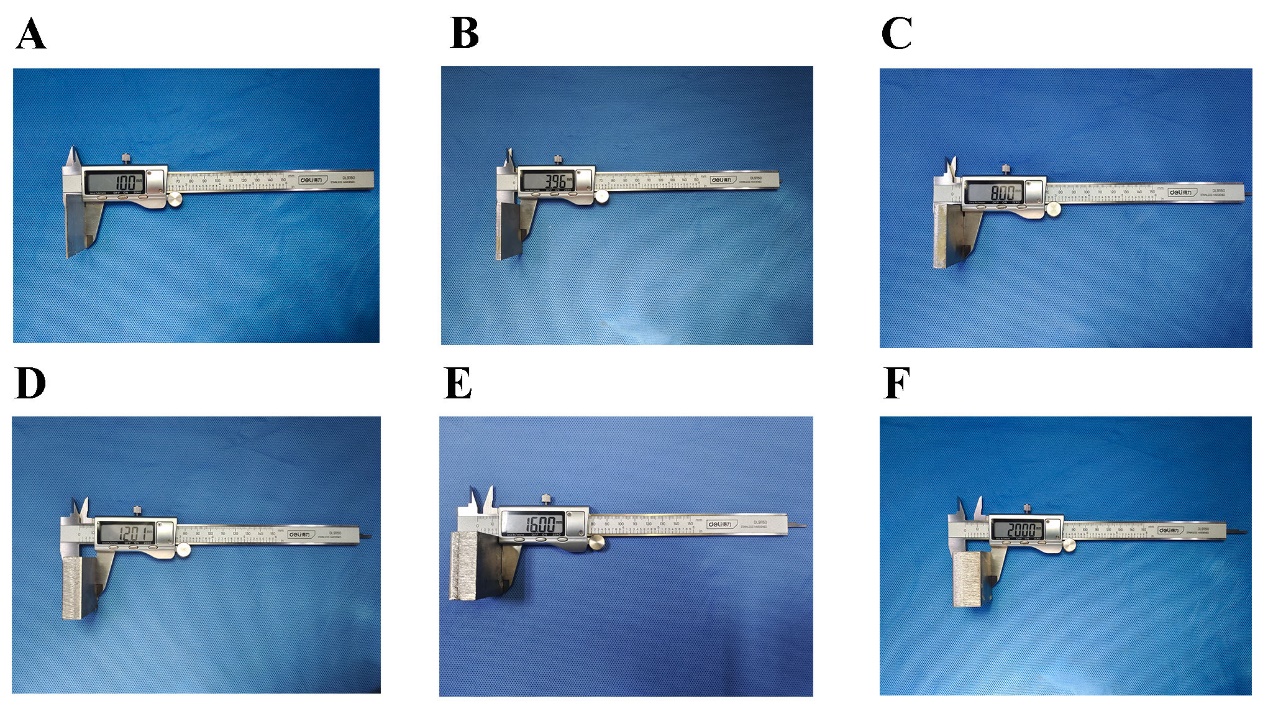


Fig.Sup.1 Iron blocks with heights of 1 mm, 4 mm, 8 mm, 12 mm, 16 mm, and 20 mm.


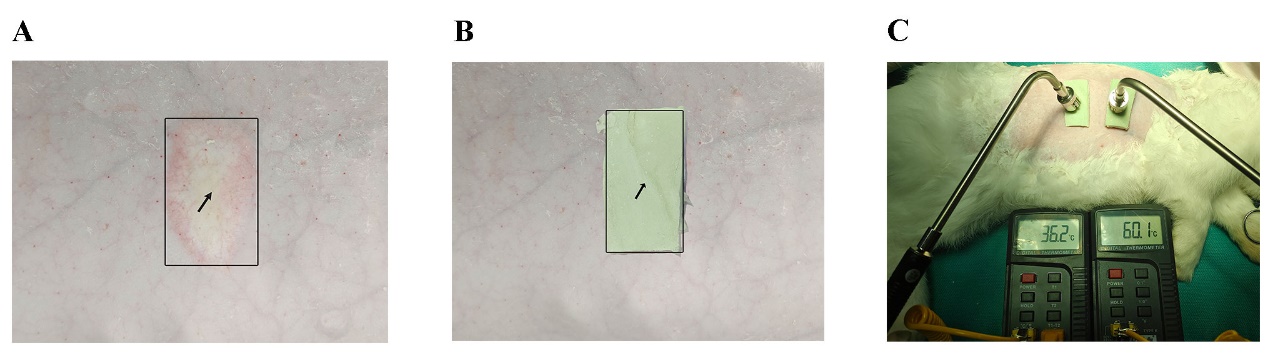


Fig.Sup.2 The location of the cement scald. A the measured area in skin. B the measured area in bone cement. C the measured method and instruments.
